# Recent temporal trends in cesarean section rates in Brazil according to the Robson classification system

**DOI:** 10.1590/0102-311XEN154024

**Published:** 2025-08-22

**Authors:** Rômulo Cesar Rezzo Pires, Maria do Carmo Leal, Antônio Augusto Moura da Silva

**Affiliations:** 1 Secretaria Municipal de Educação, São Luís, Brasil.; 2 Vice-Presidência de Ensino, Informação e Comunicação, Fundação Oswaldo Cruz, Rio de Janeiro, Brasil.; 3 Universidade Federal do Maranhão, São Luís, Brasil.

**Keywords:** Cesarean Section, Repeat Cesarean Section, Temporal Distribution, Cesárea, Recesariana, Distribuição Temporal, Cesárea, Cesárea Repetida, Distribución Temporal

## Abstract

Temporal trends in cesarean section (C-sections) rates were analyzed in Brazil and its regions using the Robson classification system. An ecological time-series study was conducted with data from the Brazilian Ministry of Health about C-section rates from 2014 to 2022. Joinpoint models were used to estimate percentage changes in C-section rate trends in the units of analysis. C-section rate in the study period was 56.4%. Robson groups with low expected C-section values (R1 to R4) represented 46% of the total rate of C-sections, with higher rates in the R2 and R4 groups. In these groups, rates increased significantly in Brazil and all its regions during the study period. However, a significant decrease in rates was observed in the R1 and R3 groups, from 2014 to 2016 in Brazil and in the North, Northeast, and Southeast regions. Despite its larger relative size among groups and greater contributions to the total C-section rate, the R5 group had a small but significant increase in rates in the Central-West Region only. Groups with higher expected values for C-sections (R6 to R10) showed a general upward trend, except for R9. The results indicate an excess of C-sections in Brazil and its regions, especially in low obstetric risk groups. Therefore, efforts to reduce unnecessary C-sections should be based on interventions to improve labor management in nulliparous women and promote vaginal birth after the procedure.

## Abstract (pt)

As tendências temporais das taxas de cesariana foram analisadas no Brasil e suas rregiões seguindo o sistema de classificação de Robson. Foi realizado um estudo ecológico de séries temporais com dados do Ministério da Saúde sobre as taxas de cesariana de 2014 a 2022. Modelos *joinpoint* foram usados para estimar mudanças percentuais nas tendências das taxas de cesariana nas unidades de análise. A taxa de cesariana no período do estudo foi de 56,4%. Os grupos de Robson com baixo valor esperado para cesariana (R1 a R4) representaram 46% da taxa total de cesarianas, com taxas mais altas nos grupos R2 e R4. Nesses grupos, as taxas de cesariana aumentaram significativamente no país e em todas as suas regiões durante o período estudado. Por outro lado, observou-se uma diminuição significativa nas taxas de cesariana nos grupos R1 e R3 de 2014 a 2016 no Brasil e nas regiões Norte, Nordeste e Sudeste. Apesar de seu maior tamanho relativo entre os grupos e maiores contribuições para a taxa total de cesarianas, o grupo R5 foi responsável por um aumento pequeno, mas significativo, na taxa de cesariana apenas na Região Centro-oeste. Os grupos com maiores valores esperados para cesarianas (R6 a R10) apresentaram tendência geral de aumento, com exceção de R9. Os resultados indicam um excesso de cesarianas no Brasil e suas regiões, especialmente em grupos de baixo risco obstétrico. Os esforços para reduzir o uso desnecessário de cesarianas devem ser baseados em intervenções para melhorar o manejo do trabalho de parto em mulheres nulíparas e promover o parto vaginal após a cesariana.

## Abstract (es)

Se analizaron las tendencias temporales en las tasas de cesáreas según el sistema de clasificación de Robson en Brasil y sus regiones. Se realizó un estudio ecológico de series temporales con datos del Ministerio de Salud sobre las tasas de cesáreas entre 2014 y 2022. Se utilizaron modelos *joinpoint* para estimar los cambios porcentuales en las tendencias de las tasas de cesáreas en las unidades de análisis. La tasa de cesáreas en el período de estudio fue del 56,4%. Los grupos de Robson con bajo valor esperado para cesáreas (R1 a R4) representaron el 46% de la tasa total de cesáreas, con tasas más altas en los grupos R2 y R4. En estos grupos, las tasas de cesáreas aumentaron significativamente en Brasil y en todas sus regiones durante el período de estudio. Por otro lado, se observó una disminución significativa en las tasas de cesáreas en los grupos R1 y R3, entre 2014 y 2016 en Brasil y en las regiones Norte, Nordeste y Sudeste. A pesar de su mayor tamaño relativo entre los grupos y su mayor contribución a la tasa total de cesáreas, el grupo R5 fue responsable de un pequeño pero significativo aumento en la tasa de cesáreas solo en la Región Centro-Oeste. Los grupos con valores esperados más altos para cesáreas (R6 a R10) mostraron una tendencia general al alza, excepto el R9. Los resultados indican un exceso de cesáreas en Brasil y sus regiones, especialmente en grupos de bajo riesgo obstétrico. Por lo tanto, los esfuerzos para reducir el uso innecesario de cesáreas deben basarse en intervenciones para mejorar el manejo del trabajo de parto en mujeres nulíparas y promover el parto vaginal tras una cesárea.

## Introduction

The number of cesarean sections (C-sections) has increased globally since the 1990s, and it is estimated that 28% of births worldwide will occur via C-section by 2030 ^1^. The World Health Organization (WHO) ^2^ highlights that C-section rates above 10% are not associated with reductions in maternal and neonatal mortality rates, and the procedure should only be performed when indicated.

Brazil has one of the highest C-section rates globally1, with a significant number of procedures considered potentially unnecessary ^3^. From 1994 to 2019, 46.4% of births in the country were via C-section, with a significant trend of 2% growth per year in the C-section rate in Brazil ^4^.

This rate is unevenly distributed throughout the country, with higher numbers in the South, Southeast, and Central-West regions. However, over the last two decades, this rate has grown rapidly in the North and Northeast regions, unnecessarily exposing women and their children to the risks of adverse outcomes during labor and birth in socioeconomically vulnerable regions. It is estimated that, by 2030, over half of births in Brazil will occur surgically, despite public policies to promote vaginal childbirth ^4^. 

Considering the above, the WHO states that efforts should focus on ensuring that C-sections are performed only when clinically indicated, rather than seeking to achieve specific rates ^5^. Based on systematic reviews ^6^ ^,^ ^7^, WHO suggests the use of the Robson classification to prospectively compare rates within and between health settings and over time ^2^ ^,^ ^7^ ^,^ ^8^. This system classifies all pregnant women into 10 groups based on their obstetric characteristics (parity, previous C-section, gestational age, labor onset, fetal presentation, and number of fetuses), informing about who women undergoing C-sections are and whether this procedure is excessively performed in any specific group. Additionally, the system can be applied prospectively, and its categories are fully inclusive and mutually exclusive - every woman admitted for delivery can be immediately classified based on these basic characteristics that are routinely collected by obstetric care providers worldwide ^9^.

In Brazil, the Robson classification was initially used in the national *Birth in Brazil* cohort ^10^ and in other descriptive hospital-based studies conducted in Brasília (Federal District) ^11^, São Paulo ^12^, Santa Catarina ^13^, and in university hospitals part of the ApiceOn network ^14^. Moreover, this classification has been employed in studies of spatial correlation with indicators of poverty ^15^, macroregional variation ^16^, and variation in socioeconomic groups ^17^.

Despite the growing body of studies using the Robson classification in Brazil, analyses regarding temporal trends of the 10 groups have not been described in the national literature, especially considering their regional differences and the use of segmented regression models. Thus, this study aimed to analyze the temporal evolution of C-section rates in Brazil and its regions according to the Robson classification, considering the hypothesis of inflection points from 2014 to 2022.

## Methods

An ecological time-series study was conducted to assess C-section trends in Brazil and its regions (North, Northeast, Southeast, South, and Central-West), according to the Robson classification.

The Robson classification system uses basic obstetric characteristics to categorize all women admitted to delivery into 10 mutually exclusive and fully inclusive groups based on the following obstetric parameters: parity; previous C-section; gestational age; labor onset; whether the C-section occurred before labor onset; fetal presentation; and number of fetuses ^9^. The categories obtained were: Group 1 (R1): nulliparous women, singleton pregnancy, cephalic presentation, ≥ 37 weeks, spontaneous onset of labor; Group 2 (R2): nulliparous women, singleton pregnancy, cephalic presentation, ≥ 37 weeks, induced labor or C-section before labor; Group 3 (R3): multiparous women (no history of C-section), singleton pregnancy, cephalic presentation, ≥ 37 weeks, spontaneous labor; Group 4 (R4): multiparous women (no history of C-section), singleton pregnancy, cephalic presentation, ≥ 37 weeks, induced labor or C-section before labor; Group 5 (R5): multiparous women, with one or more C-sections, singleton pregnancy, cephalic presentation, ≥ 37 weeks; Group 6 (R6): all breech deliveries in nulliparous women; Group 7 (R7): all breech deliveries in multiparous women (including previous C-section); Group 8 (R8): all multiple pregnancies (including previous C-section); Group 9 (R9): all abnormal presentations (including previous C-section); and Group 10 (R10): all singleton pregnancy, cephalic presentation, < 37 weeks (including previous C-section).

The R2a (induced), R2b (C-section before labor), R4a (induced), R4b (C-section before labor), R5a (only one previous C-section) and R5b (more than one previous C-section) subgroups are not available in the Brazilian Ministry of Health database and therefore could not be analyzed.

In the result description, “groups with lower expected values for C-sections” was used for the R1 to R4 groups and “groups with higher expected values for C-sections” for the R6 to R10 groups. Due to its specificities, group R5 - which includes women with previous C-sections and represents a culture called “once a cesarean, always a cesarean” - was not categorized in the subgroup with the highest risk of undergoing a C-section. Data not classified in any of the previous categories were grouped to create R11 and, in this study, it was used only as a parameter to audit data quality ^18^. Groups 1 to 4 consist of nulliparous and multiparous women without prior C-sections who have a high likelihood of vaginal birth. Group 5 includes multiparous women who have undergone previous C-sections, and groups 6 to 9 comprise women with previous C-sections or nulliparous women with babies in breech, transverse, or oblique positions, as well as women with multiple gestations. Group 10 includes women with a single cephalic fetus at less than 37 weeks, including those with previous C-sections.

The C-section rate indicator in each group of the Robson classification (number of C-sections in each group divided by the total number of births in each group) was collected from the live birth monitoring panel according to the epidemiological risk classification from 2014 to 2022 issued by the Brazilian Ministry of Health ^19^. This indicator was considered a response variable, while the regressor variable was the calendar year, whose analysis was performed for each level of aggregation adopted (Brazil and the North, Northeast, Southeast, South, and Central-West regions).

This study time frame was based on the available data from the live birth monitoring panel according to the epidemiological risk classification, as well as the high number of births without classification according to the Robson groups in the years prior to the study - including the period of system implementation in Brazil.

In addition to the C-section rate in each Robson group, the absolute and relative contributions to the total rate were calculated, as well as the relative size of each Robson group. The absolute contribution (%) is the proportion of C-sections in relation to the total obstetric population, and the relative contribution (%) is the proportion of C-sections in each Robson group in relation to the total number of C-sections. The relative size (%) is the number of births in each group in relation to the total number of births ^20^.

For trend analysis, the joinpoint regression model was used to identify possible points indicating significant changes in the trend. During modeling, it is tested whether one or more joinpoint should be added to the model. The equation of the joinpoint model used in this study was:

$$\text{ln}\left(C-\text{section rate of each group}\right)=\beta \times calendaryear+c$$ (1)

In which: *β* = calendar year coefficient, *ln* = natural logarithm, and *c* = constant (or intercept).

The slope of the line segment or annual percentage change (APC) was calculated, as well as the variation of the complete period, described as average annual percentage change (AAPC). 

The APC of C-section rates in the Robson groups between a previous calendar year “x” and the next calendar year “x+1” was:

$$\text{APC}=\left(Rate_{\left(x+1\right)}-Rate_{\left(x\right)}÷Rate_{\left(x\right)}\right)\times \text{100}$$ (2)

From Equation (1),

$Rate_{\left(x+1\right)}=e^{\beta \left(x+1\right)+c}$ and $Rate_{\left(x\right)}=e^{\beta \left(x\right)+c}$

Then,

$$APC=\left(\left(e^{\beta \left(x+1\right)+c}-e^{\beta \left(x\right)+c}\right)÷e^{\beta \left(x\right)+c}\right)\times \text{100}=\left(e^{\beta -1}\right)\times \text{100}$$

In which *e* = 2.7.

The AAPC was estimated as a weighted average of the APCs, with weights equal to the length of each line segment during the pre-specified fixed interval. An upward trend was considered when the APC/AAPC were greater than zero (positive), with the lower limits of the 95% confidence interval (95%CI) greater than zero. A downward trend was considered when the APC/AAPC were less than zero (negative), with the upper limits of the 95%CI less than zero. Rates presenting APC/AAPC equal to zero and/or with 95% CI containing zero were considered stationary.

The final selection of models was made considering the lowest values of the weighted Bayesian information criterion (WBIC), and the 95%CI were calculated by the empirical quantile method. First-order autocorrelation of errors was considered in all models analyzed. Temporal analyses were performed using the Joinpoint regression program version 5.0.1 (https://surveillance.cancer.gov/joinpoint/), and the number of points required to adjust each segment was automatically selected by the program default settings.

Since this study analyzed aggregate secondary data from an unrestricted public domain, it did not require Research Ethics Committee approval. 

## Results

### Descriptive analysis

From 2014 to 2022, 25,541,508 births were reported in Brazil, 863,477 of which were not classified in any Robson group (3.4%). C-section was the most common method of childbirth in Brazil and in its regions (except in the North), corresponding to 56.4% of all births. The highest total C-section rates were reported in the Central-West (62.9%), South (61.7%), and Southeast (59%) regions. The lowest rates were reported in the North (47.6%) and Northeast (51.7%) regions.

R5 was the group with the highest absolute and relative contributions to the total rate of C-sections in Brazil and its regions. Groups with lower expected values for C-sections (R1 to R4) accounted for about 60% of births and represented 46% of the total rate of C-sections. In these groups, the rates of C-sections were high, particularly in R2 and R4.

The total rate of C-sections at the beginning of the studied series was 57%, with a decrease until 2016 (APC = -1.3%), followed by a progressive increase until the end of the series (APC = +0.80%). The highest average percentage increases in absolute and relative contributions occurred in R5 in Brazil and the Northeast, South, Southeast, and Central-West. In the North, the highest increases occurred in R10. The highest average percentage decreases in absolute and relative contributions occurred in R9 in Brazil and its regions, except in the North (Table 1).

**Table 1:** Average annual percentage variation of the absolute and relative contributions of Robson groups to the total proportion of C-sections in Brazil and its regions, 2014-2022.

Robson groups	Brazil		North		Northeast	
	Absolute contribution	Relative contribution	Absolute contribution	Relative contribution	Absolute contribution	Relative contribution
	%	%	%	%	%	%
R1	-1.21 *	-1.46 *	-0.75	-1.67 *	-1.26 *	+2.17
R2	-1.53 *	-1.79	+1.84 *	+0.44	-0.73	-1.23 *
R3	+0.17	+0.02	+0.99 *	+0.03	+0.17	-0.70
R4	+0.52	+0.22	+3.40 *	+1.97 *	+1.67 *	+0.41
R5	+2.76 *	+2.49 *	+3.98 *	+2.98 *	+4.02 *	+2.78 *
R6	-1.32	-1.50 *	-0.69	-1.53	+0.32	-0.35
R7	+1.92 *	+1.67 *	+1.07	-0.17	+5.54 *	+4.17 *
R8	+1.32 *	+1.46 *	+1.84 *	+0.96	+2.84 *	+1.56 *
R9	-3.98 *	-4.22 *	-5.16	-7.63	-5.97 *	-5.58
R10	+1.86 *	+1.45 *	+5.74 *	+2.05 *	+3.37 *	+1.17 *
Robson groups	Southeast		South		Central-West	
	Absolute contribution	Relative contribution	Absolute contribution	Relative contribution	Absolute contribution	Relative contribution
	%	%	%	%	%	%
R1	-1.57 *	-1.13 *	-0.21	-0.52	-1.85 *	-2.34 *
R2	-2.02 *	-1.72 *	-2.35 *	-2.63 *	+0.85	+0.39
R3	-0.39	-0.23	-0.10	-0.04	-0.19	-0.54
R4	-0.02	+0.34 *	-0.63	-0.88 *	+2.59 *	+2.19 *
R5	+1.84 *	+2.08 *	+2.65 *	+2.53 *	+3.82 *	+3.35 *
R6	-0.72 *	-0.22	-1.96 *	-2.29 *	-5.88 *	-6.31 *
R7	+1.88 *	+1.82 *	+0.33	+0.02	-3.75 *	-4.14 *
R8	+1.74 *	+1.56 *	+1.48	+1.40 *	+0.91	+0.92
R9	0.00	-0.95	-5.90 *	-7.33 *	-1.15	-7.00 *
R10	+1.01 *	+1.20 *	+1.32 *	+1.24 *	+3.74 *	+3.30 *

Source: prepared by the authors.

* Significant variation.

### Trends in C-section rates in groups with the lowest expected values (R1 to R4)

In Brazil and in the Southeast, a significant downward trend was observed in C-section rates in the R1 and R3 groups in the entire study period and in the first years of the series. In the North and Northeast regions, the decreases occurred only in the initial years of the series. The Central-West showed a significant downward trend only in the study period, and a downward trend was observed in the South region only from 2014 to 2015 for the R3 group (Tables 2 and 3).

**Table 2:** Average annual percentage change (AAPC) in C-section rates in Robson groups in Brazil and its regions, 2014-2022.

Brazil				Southeast			
Robson groups	AAPC	95%CI	Trend	Robson groups	AAPC	95%CI	Trend
R1	-1.67 *	-1.41; -0.83	Decreasing	R1	-2.31 *	-2.72; -1.90	Decreasing
R2	0.90 *	0.71; 1.85	Increasing	R2	0.78 *	0.35; 1.22	Increasing
R3	-1.29 *	-1.77; -0.49	Decreasing	R3	-3.28 *	-4.34; -2.23	Decreasing
R4	2.10 *	1.78; 2.49	Increasing	R4	1.83 *	1.32; 2.50	Increasing
R5	-0.09	-0.17; 0.04	Stationary	R5	-0.23 *	-0.32; -0.09	Decreasing
R6	0.41 *	0.26; 0.57	Increasing	R6	0.32 *	0.13; 0.51	Increasing
R7	0.67 *	0.57; 0.77	Increasing	R7	0.57 *	0.29; 0.85	Increasing
R8	0.58 *	0.46; 0.70	Increasing	R8	0.26 *	0.00; 0.51	Increasing
R9	0.02	-0.03; 0.07	Stationary	R9	0.17 *	0.03; 0.31	Increasing
R10	1.12 *	0.90; 1.50	Increasing	R10	0.39 *	0.19; 0.59	Increasing
North				South			
Robson groups	AAPC	95%CI	Trend	Robson groups	AAPC	95%CI	Trend
R1	-0.07	-0.48; 0.57	Stationary	R1	-0.35	-0.96; 0.51	Stationary
R2	0.79 *	0.48; 1.09	Increasing	R2	0.32 *	0.08; 0.69	Increasing
R3	0.83 *	0.34; 1.48	Increasing	R3	-1.42	-2.58; 0.40	Stationary
R4	1.63 *	-0.39; 2.63	Increasing	R4	0.78 *	0.09; 1.47	Increasing
R5	0.04	-0.05; 0.16	Stationary	R5	0.04	-0.11; 0.26	Stationary
R6	0.05	-0.47; 0.58	Stationary	R6	0.20	-0.04; 0.44	Stationary
R7	0.39 *	0.12; 0.68	Increasing	R7	0.38	-0.05; 0.81	Stationary
R8	0.37 *	0.12; 0.74	Increasing	R8	0.49 *	0.33; 0.65	Increasing
R9	-0.27	-0.81; 0.26	Stationary	R9	0.07	-0.21; 0.35	Stationary
R10	2.25 *	1.87; 2.78	Increasing	R10	0.76 *	-0.12; 1.39	Increasing
Northeast				Central-West			
Robson groups	AAPC	95%CI	Trend	Robson groups	AAPC	95%CI	Trend
R1	-0.54	-0.89; 0.04	Stationary	R1	-1.05 *	-1.75; -0.34	Decreasing
R2	1.99 *	1.57; 2.42	Increasing	R2	1.35 *	0.98; 1.74	Increasing
R3	-0.18	-0.61; 0.38	Stationary	R3	-1.06 *	-1.80; -0.03	Decreasing
R4	3.26 *	2.31; 4.23	Increasing	R4	2.85 *	2.20; 3.62	Increasing
R5	-0.07	-0.19; 0.12	Stationary	R5	0.22 *	0.05; 0.38	Increasing
R6	1.04 *	0.57; 1.52	Increasing	R6	0.32	-0.02; 0.62	Stationary
R7	1.54 *	1.37; 1.69	Increasing	R7	0.54 *	0.14; 0.84	Increasing
R8	1.39 *	1.18; 1.60	Increasing	R8	0.38 *	0.30; 0.48	Increasing
R9	-0.02	-0.11; 0.08	Stationary	R9	-0.19	-0.39; 0.07	Stationary
R10	2.20 *	1.86; 2.75	Increasing	R10	1.76 *	0.57; 2.975	Increasing

95%CI: 95% confidence interval.

Source: prepared by the authors.

* Significant trend.

**Table 3:** Segments, joinpoints, and trends in C-section rates in Robson groups with the lowest expected values for C-sections in Brazil and its regions, 2014-2022.

Robson groups	Brazil							
	Segment 1				Segment 2			
	Period	APC	95%CI	Trend	Period	APC	95%CI	Trend
R1	2014-2016	-4.08 *	-5.11; -2.37	Decreasing	2016-2022	-0.17	-0.53; 0.40	Stationary
R2	2014-2016	-0.01	-0.83; 1.11	Stationary	2016-2022	1.21 *	0.57; 2.16	Increasing
R3	2014-2016	-6.89 *	-8.80; -3.08	Decreasing	2016-2022	0.65	-0.12; 2.27	Stationary
R4	2014-2017	1.43	-0.08; 2.84	Stationary	2017-2022	2.51 *	1.07; 4.06	Increasing
	North							
	Segment 1				Segment 2			
	Period	APC	95%CI	Trend	Period	APC	95%CI	Trend
R1	2014-2016	-4.86 *	-6.51; -1.64	Decreasing	2016-2022	1.57 *	0.94; 2.85	Increasing
R2	2014-2020	0.03	-0.54; 0.33	Stationary	2020-2022	3.09 *	1.45; 4.48	Increasing
R3	2014-2016	-8.01 *	-9.91; -5.26	Decreasing	2016-2022	3.97 *	3.21; 4.82	Increasing
R4	2014-2020	0.26	-3.67; 1.74	Stationary	2020-2022	5.83 *	1.10; 10.54	Increasing
	Northeast							
	Segment 1				Segment 2			
	Period	APC	95%CI	Trend	Period	APC	95%CI	Trend
R1	2014-2016	-3.52 *	-5.00; -0.85	Decreasing	2016-2022	0.47	-0.11; 2.14	Stationary
R2	No segment formed							
R3	2014-2016	-5.24 *	-6.95; -2.32	Decreasing	2016-2022	1.55 *	0.93; 2.51	Increasing
R4	No segment formed							
	Southeast							
	Segment 1				Segment 2			
	Period	APC	95%CI	Trend	Period	APC	95%CI	Trend
R1	2014-2018	-3.60 *	-5.45; -2.69	Decreasing	2018-2022	-1.01	-1.93; 0.93	Stationary
R2	No segment formed							
R3	2014-2018	-6.19 *	-11.30; -3.73	Decreasing	2018-2022	-0.29	-2.85; 5.55	Stationary
R4	2014-2016	0.51	-1.43; 3.41	Stationary	2016-2022	2.27	-0.34; 4.62	Stationary
	South							
	Segment 1				Segment 2			
	Period	APC	95%CI	Trend	Period	APC	95%CI	Trend
R1	2014-2016	-3.39	-5.99; 0.15	Stationary	2016-2022	0.68	-0.78; 3.50	Stationary
R2	2014-2016	-0.81	-1.85; 0.66	Stationary	2016-2022	0.70	-0.36; 2.02	Stationary
R3	2014-2016	-7.46 *	-12.10; -0.16	Decreasing	2016-2022	0.67	-2.04; 7.38	Stationary
R4	2014-2018	-0.27	-3.28; 2.53	Stationary	2018-2022	1.84	-0.93; 5.06	Stationary
	Central-West							
	Segment 1				Segment 2			
	Period	APC	95%CI	Trend	Period	APC	95%CI	Trend
R1	No segment formed							
R2	2014-2020	2.13 *	1.80; 2.91	Increasing	2020-2022	-0.94	-2.59; 0.81	Stationary
R3	2014-2016	-3.37	-6.25; 0.83	Stationary	2016-2022	-0.27	-4.00; 3.43	Stationary
R4	2014-2020	4.46 *	3.73; 6.10	Increasing	2020-2022	-1.82	-4.54; 1.80	Stationary

95%CI: 95% confidence interval; APC: annual percentage change.

Source: prepared by the authors.

* Significant trend.

In R2 and R4 C-section rates increased significantly with no joinpoints in the Northeast, Southeast, and South. In Brazil and in the North, significant increases were observed starting in 2016 and for the full study period. In the Central West, significant upward trends occurred both in the early years of the series and for the full study period (Tables 2 and 3).

### Trends in C-section rates in R5

Rates in R5 decreased significantly in the first years of the series in Brazil and its regions, except in the South and Central-West. However, in the North, Northeast, and South regions, as well as for Brazil as a whole, these rates increased significantly after 2016. In the Southeast, in addition to the decrease observed at the beginning of the series, the R5 group showed a general downward trend. Nevertheless, a slight significant increase was observed in the Central-West, when considering the entire study period (Tables 2 and 4).

**Table 4:** Segments, joinpoints, and trends in C-section rates in the Robson group 5 in Brazil and its regions, 2014-2022.

Brazil						
Period	Segment 1			Period	Segment 2		
	APC	95%CI	Trend		APC	95%CI	Trend
2014-2016	-0.93 *	-1.28; -0.27	Decreasing	2016-2022	0.19 *	0.06; 0.51	Increasing
North						
Period	Segment 1			Period	Segment 2		
	APC	95%CI	Trend		APC	95%CI	Trend
2014-2016	-1.75 *	-2.15; -1.109	Decreasing	2016-2022	0.65 *	0.51; 0.81	Increasing
Northeast						
Period	Segment 1			Period	Segment 2		
	APC	95%CI	Trend		APC	95%CI	Trend
2014-2016	-1.13 *	-1.63; -0.21	Decreasing	2016-2022	0.27 *	0.09; 0.83	Increasing
Southeast						
Period	Segment 1			Period	Segment 2		
	APC	95%CI	Trend		APC	95%CI	Trend
2014-2016	-0.94 *	-1.31; -0.32	Decreasing	2016-2022	0.01	-0.12; 0.39	Stationary
South						
Period	Segment 1			Period	Segment 2		
	APC	95%CI	Trend		APC	95%CI	Trend
2014-2016	-0.75	-1.36; 0.16	Stationary	2016-2022	0.30 *	0.01; 1.06	Increasing
Central-West							
No segment formed							

95%CI: 95% confidence interval; APC: annual percentage change;

Source: prepared by the authors.

* Significant trend.

### Trends in C-section rates in groups with the highest expected values (R6 to R10)

C-section rates showed a significant general upward trend in the R8 and R10 groups in Brazil and its regions, especially considering the last years of the historical data series (Tables 2 and 5).

In R6 and R7, rates slightly but significantly increased in Brazil and its regions, except in the South. The increases occurred in the full study period and/or in the initial years of the data series (Tables 2 and 5).

In R9, rates slightly but significantly increased only in the Southeast, with no significant change in other regions and Brazil (Table 5).

**Table 5:** Segments, joinpoints, and trends in C-section rates in Robson groups with the highest expected values for C-sections in Brazil and its regions, 2014-2022.

Robson groups	Brazil							
	Segment 1				Segment 2			
	Period	APC	95%CI	Trend	Period	APC	95%CI	Trend
R6	2014-2019	0.60 *	0.37; 1.26	Increasing	2019-2022	0.08	-0.64; 0.46	Stationary
R7	2014-2019	0.98 *	0.83; 1.23	Increasing	2019-2022	0.17	-0.33; 0.48	Stationary
R8	No segment formed							
R9	No segment formed							
R10	2014-2016	-0.60	-1.56; 1.08	Stationary	2016-2022	1.71 *	1.33; 2.90	Increasing
	North							
	Segment 1				Segment 2			
	Period	APC	95%CI	Trend	Period	APC	95%CI	Trend
R6	No segment formed							
R7	2014-2016	3.20 *	1.55; 4.52	Increasing	2016-2022	-0.52 *	-0.98; -0.24	Decreasing
R8	2014-2016	-0.69	-1.76; 0.80	Stationary	2016-2022	0.72	-0.48; 2.06	Stationary
R9	No segment formed							
R10	2014-2016	-1.92	-3.46; 0.78	Stationary	2016-2022	3.68 *	3.14; 4.66	Increasing
	Northeast							
	Segment 1				Segment 2			
	Period	APC	95%CI	Trend	Period	APC	95%CI	Trend
R6	No segment formed							
R7	2014-2016	2.23 *	1.96; 2.53	Increasing	2019-2022	0.40	-0.21; 0.93	Stationary
R8	No segment formed							
R9	No segment formed							
R10	2014-2016	0.14	-1.30; 2.50	Stationary	2016-2022	2.89 *	2.26; 4.86	Increasing
	Southeast							
	Segment 1				Segment 2			
	Period	APC	95%CI	Trend	Period	APC	95%CI	Trend
R6	No segment formed							
R7	No segment formed							
R8	No segment formed							
R9	2014-2018	0.35 *	0.07; 0.98	Increasing	2018-2022	0.00	-0.65; 0.28	Stationary
R10	2014-2018	-0.25	-1.09; 0.15	Stationary	2018-2022	1.04 *	0.63; 1.91	Increasing
	South							
	Segment 1				Segment 2			
	Period	APC	95%CI	Trend	Period	APC	95%CI	Trend
R6	No segment formed							
R7	No segment formed							
R8	No segment formed							
R9	No segment formed							
R10	No segment formed							
	Central-West							
	Segment 1				Segment 2			
	Period	APC	95%CI	Trend	Period	APC	95%CI	Trend
R6	2014-2016	1.95 *	0.51; 3.32	Increasing	2016-2022	-0.21	-1.08; 0.05	Stationary
R7	2014-2016	2.07 *	0.51; 3.43	Increasing	2016-2022	0.03	-1.17; 0.44	Stationary
R8	2014-2017	0.10	-0.34; 0.39	Stationary	2017-2022	0.55 *	0.39; 0.95	Increasing
R9	2014-2017	-0.81 *	-1.88; -0.05	Decreasing	2017-2022	0.19	-0.25; 1.26	Stationary
R10	No segment formed										

95%CI: 95% confidence interval; APC: annual percentage change.

Source: prepared by the authors.

* Significant trend.

## Discussion

### Main findings

Data from more than 25 million births from 2014 to 2022 in Brazil were analyzed temporally according to the Robson classification. C-sections were the most frequent mode of delivery in Brazil and in most of its regions. The R5 group (with previous C-sections) showed the highest contributions to the total rates at all levels of aggregation adopted. All Robson groups showed high C-section rates, especially in groups with low expected values for this procedure and predominance of upward trends in the entire study period - or at least in one of the segments created, particularly in the R2 and R4 groups. In contrast, in R1 and R3 (nulliparous and multiparous women with spontaneous labor, respectively) and R5 (with previous C-section), reductions in the rates were observed in Brazil and in most of its regions, creating a heterogeneous temporal pattern for this method.

### Interpretation

The largest obstetric population in Brazil and in the Southeast, South, and Central-West was categorized in the R5 group, which presented the highest contribution to the total C-section rate at all levels of aggregation adopted, representing 1/3 of all C-sections performed in the country. A similar result was described in a study analyzing over 8 million births from 2014 to 2016 in Brazil ^16^. A higher incidence of cases in the R5 group and their contribution to the total C-section rate is also described in other Latin American countries, such as Uruguay ^21^ and Peru ^22^; in Nordic countries ^23^ ^,^ ^24^, where the C-section rates have remained low over time due to the adoption of good obstetric practices; and in Canada ^25^. 

The R5 group consists of women with previous C-section, and interventions to reduce the number of surgeries in this group could impact the overall rate of C-sections. Knobel et al. ^16^ highlight that a decrease in C-section rates in nulliparous women (R1 and R2 groups) could lead to a decline in the size of the R5 group population and an increase in the size of the R3 and R4 groups, in which rates are lower. Another strategy would be to encourage women with a previous C-section to perform a vaginal delivery in the absence of any contraindications, following national ^26^ and international ^27^ recommendations.

The R2 and R4 groups had the highest rates in the group with conditions that were apparently favorable for vaginal delivery (R1 to R4). These rates increased significantly in Brazil and its regions during the entire study period or in at least one of the segments created during modeling. While many cases of cesarean deliveries in the R2b and R4b subgroups may have a medical indication, some may have been performed without specific indication, and the performance of C-sections at the mother’s request may be related to recent increases in rates ^28^.

C-section rates in the different Robson groups are linked to socioeconomic development indicators. In the R2 group, for example, rates are significantly higher in Brazilian states and municipalities with a high Human Development Index (HDI) ^17^ and in private health services ^10^, concentrated in large urban centers in the South and Southeast of Brazil. According to data from the United Nations Development Program (UNDP), the HDI of Brazil and the North, Northeast, Southeast, and South increased from 2012 to 2021 ^29^, which would partly explain the upward trends in C-section rates in this group. Thus, it is possible to project increases in certain Robson groups with low expectations for performing C-sections (such as R2 group) in the poorest regions of the country and a greater exposure of this obstetric population to risks involved in surgery. Similarly, another factor that could help understand the high C-section rates in specific groups would be the source of payment for the procedure. In Brazil, there is a clear difference in the composition of Robson groups and their C-section rates according to the source of payment. The two largest groups in terms of relative size in the public sector (R3 and R1) have little importance in the private sector. However, there is a clear concentration of nulliparous women in the R2b subgroup and multiparous women in the R5 group, corresponding to over 70% of C-sections performed in private sector ^10^.

Although some Robson groups (R1, R3, and R5) showed a downward trend in the proportion of C-sections in the first five years of the study in Brazil and some of its regions (North, Northeast, and Southeast), this decline was not sustained until the end of the series, which suggests the ineffectiveness of actions aimed at reducing C-sections. Hence, it is important to emphasize the need for efforts aimed at maintaining contextualized actions and public policies that encourage natural childbirth in situations in which there is no indication for C-sections, especially in groups with low expectations for the surgical procedure. Such actions can be based on successful examples of Brazilian maternity hospitals - which, based on scientific evidence, managed to safely significantly reduce the proportion of C-sections in Robson groups 1 and 3 ^30^.

In Brazil, C-section rates in the private health sector is high10 and is associated with the use of health insurances. Health insurance coverage increased significantly among people living in capitals and with a higher per capita household income (above three minimum wages) from 2013 to 2019. Moreover, among women with a health care plan, childbirth coverage is over 80% in Brazilian regions, except in the South ^31^.

The general trends of C-section rates were heterogeneous and only one joinpoint was created for each of the 60 models analyzed. Rates increased significantly in most Robson groups in Brazil (R2, R4, R6, R7, R8, and R10) and in the North (R2, R3, R4, R7, R8, and R10), Northeast (R2, R4, R6, R7, R8, and R10), and Southeast (R2, R4, R6, R7, R8, R9, and R10) from 2014 to 2022. A study conducted in Brazil with more than 24 million births from 2011 to 2017 showed that C-section rates remained stable; however, rates in Robson groups 5 and 8 increased significantly, with a downward trend in groups R2, R4, R9, and R10 ^32^. Even in scenarios where trends are decreasing, the procedure proportions remain high and higher than those recommended by the WHO for all Robson groups.

The differences found between studies can be explained by the time frame analyzed in each study, as well as by different modeling strategies adopted in the data analysis. Nevertheless, rates in the R8 group increased slightly, but significantly in Brazil and all its regions. In contrast, in R5, rates increased significantly only in the Central-West, despite its relative size and substantial contribution to the total C-section rate in Brazil.

Regarding the R5 group, a significant increase in rates was expected, especially due to its contribution to the total C-section rate in the units analyzed - as well as the results of a study conducted in Brazil, which showed a constant and linear growth of 1% per year in the R5 group contribution to the overall rate of C-sections from 2014 to 2017 ^17^. Studies indicate that even countries with low C-section rates show a positive correlation between rates in the R5 group and the total C-section rate, a fact confirmed by a significant increase in rates in the R5 group in the Central-West - the region with the highest rates in Brazil.

In Latin American, the region with the highest C-section rates worldwide (42.8%), a significant upward trend in rates was also observed for R2b, R3, R4a, R4b, R5, R8, and R10 in Uruguay ^21^ and R1, R3, R5, and R7 in Peru ^22^. These findings reinforce the heterogeneity of trends, even in spatial aggregations, and highlight that groups with conditions that are apparently favorable for vaginal childbirth also present an upward trend in C-section rates.

The trends previously described consider the evolution in C-section rates based on linear models. However, a study conducted in Brazil with over 77 million births from 1994 to 2019 showed that C-section performance is not linear, and its distribution is irregular over time ^4^. Similarly, it is believed that the creation of joinpoint occurs within Robson categories, as found in our study.

The joinpoint analysis showed a predominance of upward trends in rates in the Robson groups at the beginning of the series or as of 2016. Downward trends were concentrated mainly in the first years of the series, particularly from 2014 to 2016, followed by periods of stability. 

A pattern of obstetric care with excessive interventions and C-sections emerged in the richest regions of the country - Southeast and South - and has been reproduced in other regions, such as the Central-West and, more recently, in the North and Northeast ^4^.

Regional differences in the trends were also observed, especially in groups of low risk for C-sections (R1 to R4). In these groups, rates significantly increased in the second segments of the series in the North and Northeast. In the South and Central-West, stability was observed in the second segments. In the Southeast, there was no significant increase in any segment, only reductions in the first segment in the R1 and R3 groups, similar results to those obtained for Brazil and the North and Northeast.

Also, the sharpest and most significant decline in Brazil was observed in C-section rates in the R1 and R3 groups, especially between the beginning and median year of the historical series (2018). This sharp drop was due to significant decreases in rates in R1 and R3 in the North, Northeast, and Southeast.

In addition to the specific obstetric characteristics of each population and the care model for labor and childbirth in each region, the effect of policies can help understand the evolution of C-section rates, such as the *Appropriate Birth Project*. Ferreira ^33^ evaluated the post-implementation effect of this project on the C-section rate from 2014 to 2018. When analyzing the results based on the Robson classification, the reduction in R1, R2, R3, and R4 combined was of 58.31% to 52.83% in public health facilities and of 65.69% to 57.85% in private health facilities participating in the project, which would partly explain the decreases observed during the period.

Another important aspect is that, in Brazil, there was an upward trend in C-sections in all Robson groups in public healthcare facilities, while in the private sectors there was a slight downward trend from 2014 to 2020 ^34^.

Breech presentation (R6 and R7 groups), multiple pregnancy (R8 group), oblique or transverse lie (R9 group), and preterm birth (R10 group) have a relatively small contribution to the overall C-section rates and, in fact, represent a reasonably constant proportion in each population ^35^, accounting for less than 15% of births in the study.

In R8 and R10, rates increased significantly at all levels of the analysis. It is plausible to assume that women with previous preterm birth due to obstetric intervention have an even higher risk of recurrence due to the effects of obstetric care organization and the mother’s choice of the same type of childbirth, particularly C-sections ^36^. 

Regarding the ubiquitous upward trend in C-section rates in the R8 group, Cardoso-dos-Santos et al. ^37^ reported an upward trend in the twinning rate from 2001 to 2014, both for the country and for all states in the Southeast, South, and Central-West. This is possibly due to late pregnancy and greater access to assisted reproduction techniques. As a result, a higher number of C-sections has been observed in this group. 

### Strengths and limitations

This study assessed data of over 25 million live births in Brazil and its regions, identifying specific temporal patterns considering the country’s heterogeneity and the creation of joinpoint in the series. Estimates described here are considered consistent and conditional to the coverage of Brazilian Information System on Live Births (SINASC, acronym in Portuguese), which has been homogeneous and high in the country ^38^. Moreover, only 3.4% of the total obstetric population was not classified in any Robson category, and this value is considered a useful parameter for data quality assessment ^18^. Another important quality parameter assessed was the relative size of the R9 group (oblique or transverse presentation), whose value was within the suggested range (0.2 to 0.6%). However, this type of birth must be uncommon in the population and its proportion is close to 100% ^39^.

Nevertheless, some limitations were observed. Despite the robustness and effectiveness of the Robson classification in monitoring C-sections, the system disregards other important variables such as maternal age, clinical indication for C-section, and source of payment, which did not enable analysis adjusted for these variables. In this study, subdivisions into groups R2 (2a and 2b), R4 (4a and 4b), and R5 (5a and 5b) were not used, since these were not available in the database. Such analysis would provide more specific information on the number of C-sections performed and whether they were performed before labor.

Furthermore, the use of secondary data collected in a non-standardized manner throughout the country could generate information bias due to the incorrect classification of Robson’s groups. Therefore, the frequency would be overestimated in some groups and underestimated in others, modifying estimates of the trends found. The ecological design does not enable an accurate identification of determinants that change the trend in C-section rates, which was another limitation in the interpretation of the results.

## Final considerations

High C-section rates were observed in Brazil, especially among obstetric populations at low risk for the procedure. This suggests that many C-sections may have been performed without clinical indication, with subsequent increases in C-section rates. Varied trend patterns were observed over time in the different units of analysis, demonstrating heterogeneity and predominance of growth patterns in rates over the entire period or starting in the second segments generated by the modeling strategy. Thus, efforts to reduce unnecessary C-sections should be based on interventions to reduce the number of primary and repeat C-sections. In Japan, for instance, a significant decrease in C-section rates was achieved by reducing the contribution to C-section rates in the group of nulliparous women with spontaneous or induced labor (R1 and R2a groups) and women with a previous C-section (R5). Interventions can be combined with a focus on health professionals and the management of health services and systems, preparation for natural birth during prenatal care, access to different environments for birth, support for vaginal birth after previous C-section, and ongoing support for women during labor and delivery evidence-based actions.
